# Narrative Organization Deficit in Lewy Body Disorders Is Related to Alzheimer Pathology

**DOI:** 10.3389/fnins.2017.00053

**Published:** 2017-02-08

**Authors:** Murray Grossman, David J. Irwin, Charles Jester, Amy Halpin, Sharon Ash, Katya Rascovsky, Daniel Weintraub, Corey T. McMillan

**Affiliations:** ^1^Departments of Neurology, University of PennsylvaniaPA, USA; ^2^Departments of Psychiatry, University of PennsylvaniaPA, USA

**Keywords:** Lewy body disease, narrative comprehension, frontal lobe, cerebrospinal fluid, amyloid, tau

## Abstract

**Background:** Day-to-day interactions depend on conversational narrative, and we examine here the neurobiological basis for difficulty organizing narrative discourse in patients with Lewy body disorders (LBD).

**Method:** Narrative organization was examined in 56 non-aphasic LBD patients, including a non-demented cohort (*n* = 30) with Parkinson's disease (PD) or PD-Mild Cognitive Impairment PD-MCI,) and a cohort with mild dementia (*n* = 26) including PD-dementia (PDD) and dementia with Lewy bodies (DLB), with similar age and education but differing in MMSE (*p* < 0.001). We used a previously reported procedure that probes patients' judgments of the organization of brief, familiar narratives (e.g., going fishing, wrapping a present). A subgroup of 24 patients had MRI assessment of regional gray matter (GM) atrophy and cerebrospinal fluid (CSF) levels of biomarkers for Alzheimer's disease (AD) pathology, including beta amyloid (Aβ), total-tau (*t*-tau), and phosphorylated-tau (*p*-tau).

**Results:** Mildly demented LBD patients had a significant deficit judging narratives compared to non-demented patients, but this deficit was not correlated with MMSE. Regression analyses instead related narrative organization to regions of frontal GM atrophy, and CSF levels of Aβ and *t*-tau associated with presumed AD pathology in these frontal regions.

**Conclusion:** These findings are consistent with the hypothesis that CSF markers of AD pathology associated with frontal regions play a role in difficulty organizing narratives in LBD.

## Introduction

Daily conversations are critical for day-to-day safety and quality of life. Conversations consist of narratives or a thematically organized description of events and thoughts. Cognitive deficits on neuropsychological measures have been reported in patients with Parkinson's disease (PD) and related disorders (Hessen et al., 2016), but functional deficits such as limited narrative processing during a conversation are arguably as important since vague and tangential communication can have a profound impact on safety and quality of life (Kempster et al., 2010; Rosenthal et al., 2010). Previous work has shown deficits in non-aphasic PD patients' narrative expression primarily due to an organizational deficit (Ash et al., 2011). In the present study, we examined the neurobiologic basis for difficulty understanding narrative organization.

PD is primarily characterized by motor features, and current consensus criteria do not exclude a PD diagnosis when cognitive impairment is present at the time of motor onset (Postuma et al., 2015). Over 20% of PD patients have cognitive difficulties at presentation and 10% meet criteria for mild cognitive impairment (PD-MCI; Litvan et al., 2012; Weintraub et al., 2015). Over the course of disease, up to 80% develop dementia (PDD; Aarsland et al., 2003; Emre et al., 2007). In dementia with Lewy bodies (DLB), cognitive difficulties resembling PDD precede motor features or occur within 1 year of motor onset (McKeith et al., 2005). We refer to these variants collectively as Lewy body disorders (LBD; Toledo et al., 2016).

Despite varying degrees of cognitive difficulty, neuropathological studies link LBD to a shared pathology, the abnormal aggregation of α-synuclein (α-syn) into Lewy body inclusions (Irwin et al., 2012a; Goedert et al., 2013). Moreover, many LBD patients have histopathological features of Alzheimer's disease (AD) at autopsy, including amyloid-beta (Aβ) plaques and neurofibrillary tangles composed of tau (Braak and Braak, 1990; Irwin et al., 2012a). Aβ pathology has been related to cognitive impairment in LBD, including amyloid-PET and cerebrospinal fluid (CSF) Aβ_1−42_ (Montine et al., 2010; Siderowf et al., 2010; Gomperts et al., 2013; Bäckström et al., 2015; McMillan and Wolk, 2016). The relationship between CSF tau and cognition in LBD has been less clear: Some associate cognitive difficulty with elevated CSF tau (Hall et al., 2016; Kang et al., 2016), but this is not universally confirmed (Terrelonge et al., 2016). In a recent autopsy-based study, we found that AD-related Aβ and tau pathology are each associated with more rapid clinical decline and earlier presentation of cognitive difficulties (Irwin et al., 2017).

**Figure 1:**
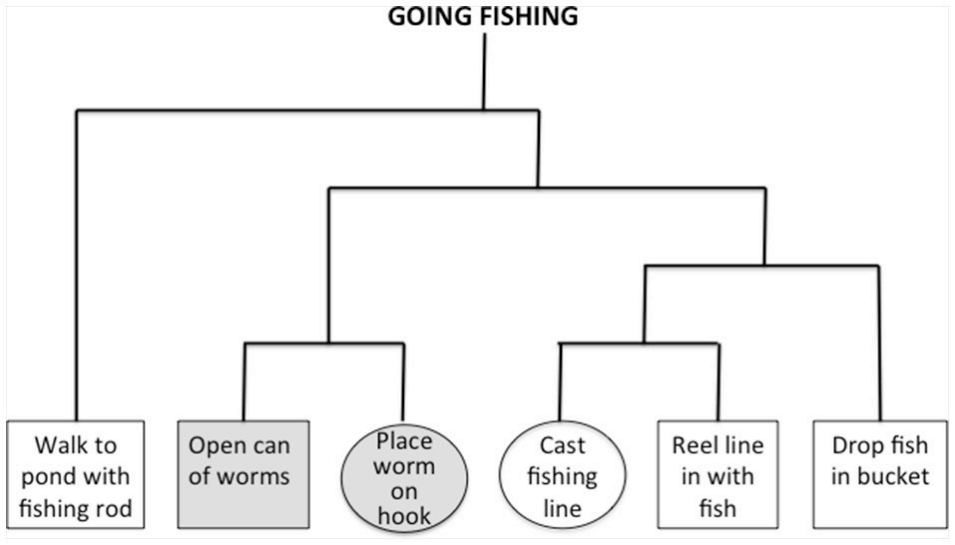
**Example of hierarchal structure for the script “going fishing.”** Empirical judgments of an independent cohort of healthy subjects are used to assess associativity strengths between all pairs of events in a narrative, and these are used to construct the hierarchical structure depicted in the figure. Events from the same cluster have a higher associativity strength than events from different clusters. We identified triplets of overlapping within-event and between-event clusters, and these did not involve initial or terminal events. In the figure, consecutive events with a gray background are within the same cluster, and circled events are from different clusters. Impaired narrative organization is reflected by difficulty judging the order of between-cluster events compared to within-cluster events. All probed pairs of events occur consecutively within a narrative, and thus greater difficulty judging different-cluster events compared to within-cluster events cannot be attributed easily to ordering difficulty.

Rare studies have related AD pathology in LBD to functional impairments impacting quality of life. Here, we investigated how AD biomarkers in LBD are related to narrative processing. We used a previously-reported procedure that probes familiar activities such as “going fishing.” In non-aphasic patients with frontotemporal degeneration (FTD), narrative comprehension is impaired because of difficulty understanding the organization of a narrative (Farag et al., 2010; Figure 1), and this has been linked to dorsolateral and inferior prefrontal areas. This appeared to be a relatively selective deficit, because we did not find a similar association in patients with typical amnestic Alzheimer's disease (Farag et al., 2010). These frontal areas are atrophic in LBD (Cordato et al., 2000; Burton et al., 2004), and Aβ and tau pathology is found in these frontal regions (Irwin et al., 2012a; Howlett et al., 2015). Based on these findings, we hypothesized that narrative organization would be impaired in LBD, related to frontal atrophy and a CSF profile associated with AD pathology.

## Methods

### Participants

We assessed 56 non-aphasic, native English-speakers with a clinical syndrome consistent with LBD. Patients were diagnosed by board-certified neurologists using published consensus criteria in a weekly consensus conference (Hughes et al., 1992; McKeith et al., 2005; Emre et al., 2007; Litvan et al., 2012). A summary of clinical features is provided in Table 1. Aphasia was excluded by performance on a structured mental status exam (Libon et al., 2011). We also excluded patients with other neurodegenerative diseases, other neurologic conditions such as stroke or hydrocephalus, a primary psychiatric disorder, or medical conditions causing cognitive difficulty.

**Table 1:** **Mean (±S.D.) demographic and clinical characteristics of Lewy body-related disorder patients**.

**Group (*n*)**	**Age (Years)**	**Education (Years)**	**UPDRS parts 1/2/3**	**MMSE^a^ (Max = 30)**	**BNT (Max = 30)**	**FAS (#/Min)**	**PVLT (Max = 9)**
Non-demented (30)	68.7 (6.2)	17.1 (2.3)	1.7/9.6/20.6	28.6 (1.6)	27.2 (2.5)	47.5 (12.8)	6.9 (2.2)
PD (12)	67.4 (6.5)	16.8 (2.8)	0.8/5.3/21.4	29.0 (1.0)	27.3 (2.2)	47.7 (12.0)	7.2 (2.1)
PD-MCI (18)	69.6 (6.0)	17.2 (2.0)	2.0/11.7/20.3	28.4 (2.0)	27.0 (3.4)	47.5 (14.0)	6.6 (2.3)
Demented (26)	71.5 (8.9)	16.0 (2.2)	2.1/8.3/10.8	25.0 (4.2)	25.9 (3.0)	26.3 (12.9)	3.6 (2.1)
PD-Dementia (14)	74.4 (8.5)	15.9 (2.3)	3.0/14.6/24.6	25.1 (2.6)	26.3 (2.9)	27.4 (13.5)	3.7 (2.3)
Dementia with Lewy Bodies (12)	68.2 (8.5)	16.3 (2.3)	3.3/9.8/14.4	24.9 (5.2)	25.5 (3.4)	25.3 (13.3)	3.5 (2.0)
Normal CSF Aβ_1−42_ (non-demented = 10, demented = 8)	66.7 (7.8)	16.6 (2.4)	2.2/10.8/22.2	28.3 (1.6)	27.9 (2.0)	39.1 (11.7)	6.1 (2.1)
Low CSF Aβ_1−42_ (non-demented = 2, demented = 4)	69.0 (9.0)	14.3 (2.3)	3.5/13.5/27.8	26.7 (1.6)	25.3 (1.7)	37.4 (12.5)	3.3 (1.5)

a *ANOVAs showed that MMSE differs between non-demented patients and demented patients (p < 0.001) and between amyloid-positive and amyloid-negative groups (p = 0.046). MMSE was not available in two non-demented patients and six demented patients, and all of these patients were in the amyloid-negative group. BNT, Boston Naming Test. FAS, letter-guided category naming fluency of each letter (i.e., F, A, and S) for 1 min. PVLT, Philadelphia Verbal Learning Test delayed recall*.

To evaluate impaired narrative organization, we subdivided patients into two groups: A non-demented group [*n* = 30; mean (±S.D.) MMSE = 28.6 (±1.6)] composed of 12 patients with PD-normal cognition (PD-NC) and 18 PD-MCI patients; and a mildly demented group [*n* = 26; mean (±S.D.) MMSE = 25.0 (±4.2)] including 14 PDD patients and 12 DLB patients. We focused on mildly demented patients to select cases that were most comparable to the non-demented patients while allowing us to study a broad spectrum of patients with LBD. Groups were comparable for age and education and by definition, non-demented and demented LBD patients differed in MMSE (*t* = 4.21; *p* < 0.001) and performance on a verbal measure delayed memory recall (*t* = 4.56; *p* < 0.001; Table 1). Table 1 also shows that groups did not differ in performance on a confrontation naming test (*p* > 0.3) or on a letter-guided category naming fluency measure of executive functioning (*p* > 0.1). Twenty-four patients (12 non-demented, 12 demented, matched demographically with their respected groups, all *p* > 0.1) also underwent CSF sampling and analysis and MRI. Table 1 also shows that groups with *low A*β_1−42_ and *normal A*β_1−42_ CSF levels were matched for age and education, and differed marginally in MMSE (*t* = 2.16; *p* < 0.05). Some of these patients contributed to a previous report where we described latency performance rather than accuracy (see below; Ash et al., 2012). Fifteen community-dwelling healthy seniors served as a normal cognitive reference group for LBD patients. All subjects participated in an informed consent procedure approved by the University of Pennsylvania Institutional Review Board.

### Cognitive materials

Using a reported procedure (Farag et al., 2010), we probed 22 narratives describing familiar activities, each containing six routine events. Briefly, a cohort of young control subjects were presented with the six events of each script typed on separate pieces paper, and asked to order the events. The subjects then were asked to cluster the events of each script into closely associated clusters. Based on these clusters, we derived associativity strengths between pairs of events and formed these into a hierarchical tree. Figure 1 illustrates an example of an empirically-derived, hierarchically-organized tree structure for the narrative “going fishing.” To examine narrative organization, we identified event triplets where one consecutive pair of events came from the same hierarchical cluster (within-cluster, gray events in Figure 1), and another consecutive pair (where one event was shared with the within-cluster pair) came from two different hierarchical clusters (different-cluster, circled events in Figure 1). Greater difficulty judging pairs of consecutive events from different clusters compared to the same cluster would be consistent with a deficit understanding narrative organization.

We generated four types of experimental stimuli in a counterbalanced 2 × 2 design presented in a pseudo-random order. Each stimulus was comprised of two events (e.g., “open can of worms,” “place worm on hook”). The experimental design included a factor for Narrative Organization (within-cluster, different-cluster) and a factor for Order (correct order, incorrect order). We presented one instance of each type of stimulus pair from each of the 22 scripts, resulting in 88 stimuli. There was no difference in overall ordering accuracy across LBD groups [*F*_(3, 52)_ = 2.12; *p* > 0.1], and we report below the critical judgments of correctly-ordered adjacent events from the same cluster compared to correctly-ordered adjacent events from different clusters. We also presented filler material composed of 44 correctly-ordered pairs of non-adjacent different-cluster events from these triplets and incorrectly-ordered pairs of these non-adjacent different-cluster events. For each stimulus, the narrative title was presented at the top of a computer screen and a pair of events was displayed below this. Participants took as much time as needed to judge event order accuracy for the named script. Responses were recorded by pressing one of two buttons on the computer keyboard. Prior to the experiment, participants were given a practice run with six judgments, and incorrect answers were explained by the experimenter. All participants understood instructions.

### CSF analyses

Methods for acquiring and storing CSF Aβ_1−42_, *t*-tau, and *p*-tau were described previously (Shaw et al., 2009; Irwin et al., 2012b). Briefly, CSF samples were obtained by lumbar puncture using a 22-gauge spinal needle as described in the Alzheimer's Disease Neuroimaging Initiative (ADNI) procedures manual (http://www.adni-info.org/). CSF was divided into aliquots (0.25 mL) and stored in bar code-labeled polypropylene vials at −80°C. Aβ_1−42_ was measured using the multiplex xMAP Luminex platform (Luminex Corp, Austin, TX) with Innogenetics (INNO-BIA AlzBio3; Ghent, Belgium; for research use only reagents) immunoassay kit-based reagents (Shaw et al., 2009). Full details for the combination of immunoassay reagents and analytical platform employed in the present study are explained elsewhere (Irwin et al., 2012b). Reliability studies (http://www.adni-info.org) show high reproducibility for this biomarker, with <10% variance. In line with previous studies (Shaw et al., 2009), CSF Aβ_1−42_ was analyzed as a binary measure employing a level of ≤192 pg/mL as a cutoff, resulting in two subgroups of patients: *low A*β_1−42_ patients (≤192 pg/mL, *n* = 11) with CSF levels that are associated with AD pathology (Shaw et al., 2009; Irwin et al., 2012b), and *normal A*β_1−42_ patients (>192 pg/mL, *n* = 26) with CSF levels that are not associated with AD pathology. The clinical features associated with these groups are summarized in Table 1. CSF *t*-tau and *p*-tau were assessed parametrically with correlation and linear regression, and the model included age, MMSE, and CSF Aβ.

### Statistical analyses

Behavioral analyses used analysis of variance (ANOVA) with a group X narrative organization design, with follow-up *t*-tests and correlations to help interpret the results. We accepted as significant only statistical results exceeding *p* < 0.05. Linear regressions were used to relate behavioral performance to CSF analyte levels.

### Imaging analyses

Methods for acquisition and preliminary analysis of MRI imaging were described previously (Tustison et al., 2014; McMillan and Wolk, 2016). Briefly, high-resolution volumetric T1-weighted MRI was obtained within 9 months of behavioral testing. Imaged patients matched the overall group in age, education, MMSE and gender (all *p* > 0.6). Exclusion from MRI was related to health and safety (e.g., metallic implants, shrapnel, claustrophobia), intercurrent medical illness, transportation difficulty, and lack of interest in participating in an imaging study. MRI volumes were acquired using an MPRAGE sequence from a SIEMENS 3.0T Trio scanner with an 8-channel head coil and the following acquisition parameters: repetition time = 1620 ms; echo time = 3.87 ms; slice thickness = 1.0 mm; flip angle = 15°; matrix = 192 × 256, and in-plane resolution = 1.0 × 1.0 mm, or using a GE 1.5T scanner with 1.2-mm slice thickness and a 144 × 256 matrix. Images from both scanners were de-formed into a standard local template space with a 1-mm^3^ resolution. Whole-brain MRI volumes were preprocessed using an established pipeline with Advanced Normalization Tools (Tustison et al., 2014). Briefly, ANTs performs a diffeomorphic deformation of each individual dataset into a standard local template space. Template-based priors guide GM segmentation and compute GM probability, reflecting a quantitative measure of GM density. Resulting images are warped into MNI space, smoothed using a 4 mm full-width half-maximum Gaussian kernel and downsampled to 2 mm resolution. Permutation-based imaging analyses were performed with threshold-free cluster enhancement (TFCE; Smith and Nichols, 2009) using the randomize tool in FSL (http://fsl.fmrib.ox.ac.uk/fsl/fslwiki).

In the core analysis, GM density was compared in LBD patients relative to healthy seniors (an independent group of 32 healthy seniors with imaging who matched the patient group for education, age, and gender; all *p* > 0.2) using a permutation-based (*n* = 10,000 permutations) *t*-test with the randomize tool in FSL (http://fsl.fmrib.ox.ac.uk/fsl/fslwiki). Analysis was restricted to voxels containing high probability GM (>0.3) using an explicit mask generated from the average GM probability map of all participants. We report clusters (minimum of 50 adjacent voxels) that survived a *p* < 0.05 threshold-free cluster enhancement (TFCE; Smith and Nichols, 2009) threshold with family-wise error (FWE) correction after covarying for age and scanner used to collect data.

**Figure 2:**
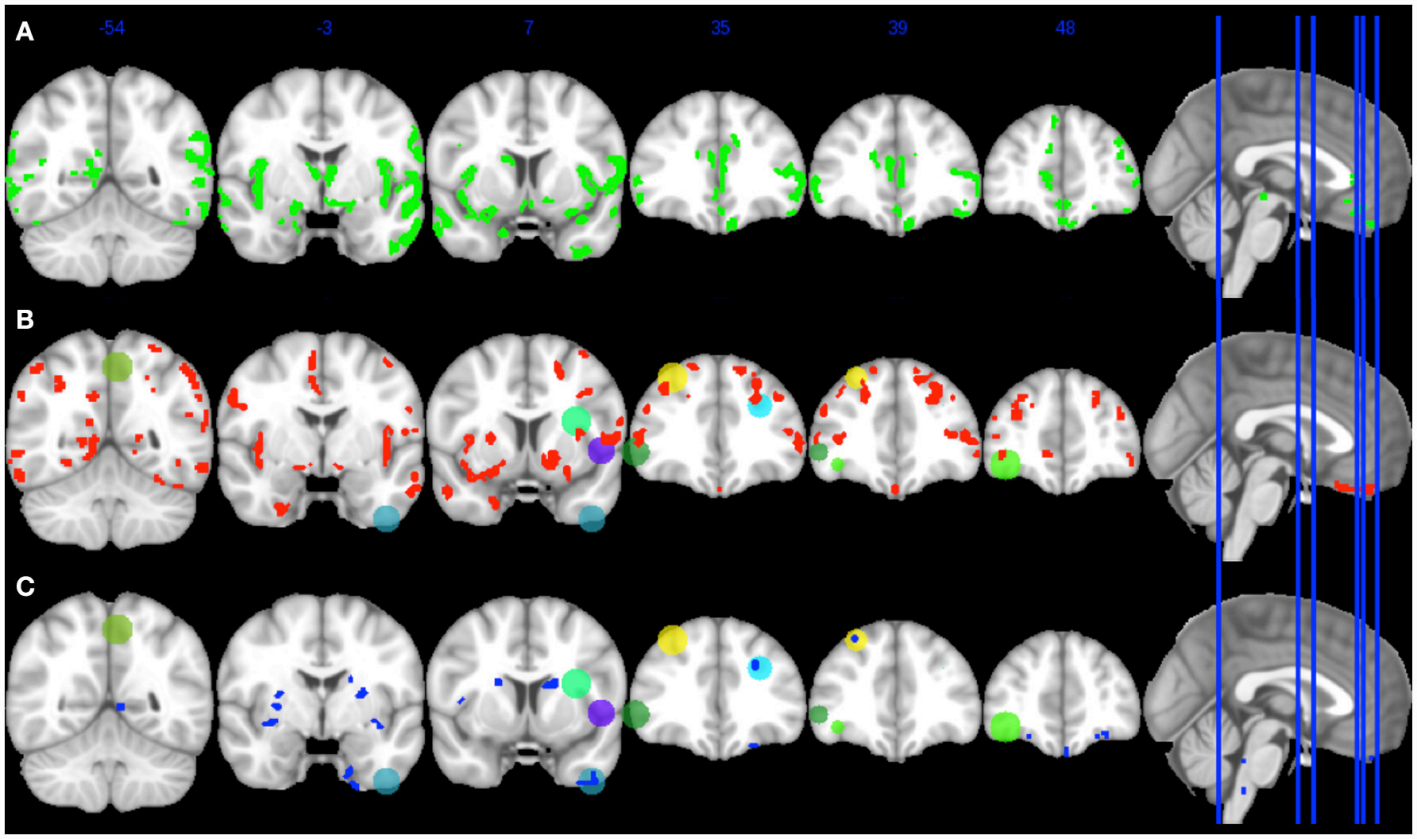
**Gray matter atrophy in Lewy body spectrum disorders, and relationship of gray matter atrophy in regions critical for narrative judgments to cerebrospinal fluid analytes**. Panel **(A)**: Significant gray matter atrophy in Lewy body spectrum disorder patients compared to controls. Green blobs indicate gray matter atrophy at *p* < 0.05 FWE-corrected. Coronal slice *y*-axis is indicated above each image and illustrated on the sagittal image at the right. Panel **(B)**: Regions of significant gray matter atrophy in Lewy body spectrum disorder patients with low CSF Aβ compared to normal CSF Aβ (red blobs), and overlap of these areas with hypothesized regions of interest related to narrative organization from Farag et al. (2010; see color code in Table 2). Panel **(C)**: Gray matter regions significantly related to CSF total-tau (blue blobs), and overlap of these areas with regions of interest related to narrative organization from Farag et al. (2010; see color code in Table 2).

To evaluate the relationship of narrative judgments with GM atrophy and CSF biomarkers, we performed three region-of-interest (ROI) analyses. These hypothesized ROIs were defined using a prior study relating narrative organization performance to GM atrophy in AD or FTD (six ROIs) and to fMRI in healthy adults (two ROIs; Farag et al., 2010). For each ROI, we generated a 10 mm radius sphere around each peak voxel (see Table 2 for peak locations). In each experimental regression investigating the relationship between narrative judgments and imaging, we performed permutation-based (*n* = 10,000 permutations) analyses at *p* < 0.05 and *k* = 50 in analyses illustrated in Figures 2A,C, and *k* = 15 in Figure 2B, using the randomize tool in FSL, including covariates for age at time of MRI and scanner, and one-tailed statistical tests were used because of our *a priori* hypotheses.

**Table 2:** **Gray matter atrophy in lewy body spectrum disorders, and relationship of gray matter atrophy in regions critical for narrative judgments to cerebrospinal fluid analytes**.

	**Beta weight**	***r*****-square**	***p*****-value**	**Peak coordinate**		
				**x**	**y**	**z**
**PEAK COORDINATES OF SPHERES RELATED TO NARRATIVE ORGANIZATION, AND REGRESSIONS RELATING THESE SPHERES TO PERFORMANCE ON DIFFERENT-CLUSTER JUDGMENTS^a^**						
Left dorsolateral prefrontal	0.06361	0.121	0.047	−26	29	28
Left inferior frontal (fMRI)	0.1028	0.251	0.005	−52	12	−1
Right inferior frontal (fMRI)	0.05925	0.124	0.040	54	31	−2
Right inferolateral frontal	0.06695	0.151	0.027	42	48	−10
Left anterolateral temporal	0.05709	0.159	0.030	−46	2	−45
Left medial parietal	0.1123	0.276	0.004	−10	−55	53
Left inferior frontal	0.05624	0.112	0.055	−36	9	18
Right superior frontal	0.05592	0.093	0.074	30	32	46
	**Color**		**A**β **overlap^b^ (voxels)**	**A**β **overlap^b^ (%)**		
**OVERLAP OF SPHERES RELATED TO NARRATIVE ORGANIZATION WITH GRAY MATTER ATROPHY RELATED TO LOW CSF A**β						
Left dorsolateral prefrontal	Light blue		12	16		
Left inferior frontal (fMRI)	Purple		22	30		
Right inferior frontal (fMRI)	Light green		17	22		
Right inferolateral frontal	Dark green		13	17		
Left anterolateral temporal	Middle blue		0	0		
Left medial parietal	Middle green		0	0		
Left inferior frontal	Green-blue		12	16		
Right superior frontal	Yellow		15	19		
	**Color**		**Regression overlap (voxels)**	**Regression overlap (%)**		
**OVERLAP OF SPHERES RELATED TO NARRATIVE ORGANIZATION WITH GRAY MATTER ATROPHY RELATED TO TOTAL TAU**						
Left dorsolateral prefrontal	Light blue		21	27		
Left inferior frontal (fMRI)	Purple		0	0		
Right inferior frontal (fMRI)	Light green		0	0		
Right inferolateral frontal	Dark green		0	0		
Left anterolateral temporal	Middle blue		18	23		
Left medial parietal	Middle green		0	0		
Left inferior frontal	Green-blue		0	0		
Right superior frontal	Yellow		4	5		

a *Two peaks were taken from fMRI activations during task performance in healthy controls, and six regions from regressions of task performance with patient gray matter atrophy from Farag et al. (2010)*.

b *Overlap refers to the number of shared voxels and the % shared volume that regressions in PD spectrum disorders overlap with the 10 mm spheres created around peak coordinates associated with narrative structure judgments in Farag et al. (2010)*.

In the first ROI analysis, we assessed how LBD narrative performance relates to GM atrophy in the hypothesized ROIs using univariate regressions relating mean GM density in each ROI to a behavioral difference score (different-cluster minus within-cluster judgments). This analysis and the regressions below used the randomize tool in FSL with 10,000 permutations at *p* < 0.05 with *k* = 50. Here and below, we covaried for age at time of MRI and scanner.

In the second analysis, we assessed the role of Aβ in narrative judgments. A *t*-test identified regions of GM atrophy in LBD with low CSF Aβ relative to LBD with normal CSF Aβ, using an explicit mask consisting of regions associated with GM atrophy in all LBD relative to controls, with *k* = 15. We evaluated the overlap between regions associated with low CSF Aβ and the hypothesized ROIs.

Third, we examined the relationship between areas of GM density associated with elevated CSF tau and narrative organization difficulty. We identified areas of GM atrophy significantly related to elevated CSF *t*-tau or *p*-tau using regression analyses with *k* = 50, masked by areas of GM atrophy in all LBD patients compared to controls. We then identified areas of GM atrophy associated with CSF tau that overlapped with the hypothesized ROIs.

## Results

### Behavioral results

An ANOVA with a group (non-demented, demented) X narrative organization (within-cluster, different-cluster) design revealed a significant main effect for narrative organization [*F*_(1, 54)_ = 17.60; *p* < 0.001] and a significant group X narrative organization interaction effect [*F*_(1, 54)_ = 11.76; *p* = 0.001]. For judgments of different-cluster events, demented patients [mean (±S.D.) % correct = 82.69 (±15.0)] performed worse than non-demented patients [mean (±S.D.) % correct = 92.27 (±13.7)] [*t*_(54)_ = 2.49; *p* = 0.006]. By comparison, for judgments of within-cluster events, non-demented patients [mean (±S.D.) % correct = 93.18 (±17.3)] and demented patients [mean (±S.D.) % correct = 91.78 (±10.2)] were equally accurate. Demented patients had greater difficulty with different-cluster events compared to their own judgments of within-cluster events [*t*_(25)_ = 4.14; *p* < 0.001]. We also examined each individual's difference score for within-cluster and different-cluster events, and found a greater difference in demented compared to non-demented patients [*t*_(54)_ = 3.43; *p* = 0.001]. Although groups differed in MMSE, we did not find a correlation between MMSE and the difference score [*r*_(20)_ = −0.14; ns], nor between MMSE and different-cluster judgments [*r*_(20)_ = 0.53; ns]. These findings indicated that LBD patients with dementia have relative difficulty understanding narrative organization. Since there was a poor correlation between performance and overall dementia severity, we examined other factors that may be contributing to difficulty understanding narrative organization.

### CSF analyses

An ANOVA with a group (low-Aβ, normal-Aβ) X narrative organization (within-cluster, different-cluster) design found a significant main effect for narrative organization [*F*_(1, 22)_ = 15.88; *p* = 0.001] and a significant group X narrative organization interaction [*F*_(1, 22)_ = 8.35; *p* = 0.009]. The interaction remained significant after covarying for MMSE [*F*_(1, 19)_ = 5.62; *p* = 0.029]. LBD with low Aβ had greater difficulty judging different-cluster events compared to their own judgments of within-cluster events [*t*_(5)_ = 3.21; *p* = 0.024], and the difference score for within-cluster and different-cluster judgments was significantly greater in patients with low-Aβ compared to normal-Aβ [*t*_(22)_ = 2.89; *p* = 0.009]. Although analyses of Aβ are typically treated dichotomously, we performed exploratory analyses examining the impact of Aβ on narrative organization that treat Aβ as a continuous variable because of the small number of abnormal Aβ values. Regression confirmed that different-cluster judgments are associated with CSF Aβ level [*F*_(1, 28)_ = 5.16; *p* = 0.031], accounting for 15.6% of the variance (β = 0.394). Here, we found a correlation indicating that accuracy for different-cluster judgments decreased as CSF Aβ levels decreased [*r*_(28)_ = 0.39; *p* = 0.031].

Different-cluster judgments were correlated with CSF *t*-tau levels [*r*_(28)_ = −0.59; *p* < 0.001]. Regression showed that different-cluster judgments are associated with CSF *t*-tau level [*F*_(1, 28)_ = 14.93; *p* < 0.001], accounting for 34.8% of the variance (β = −0.590). Age, MMSE, and CSF Aβ level did not contribute. Accuracy judging different-cluster events decreased as CSF *t*-tau levels increased [*r*_(24)_ = −0.56; *p* = 0.004]. Only the result for *t*-tau survived in a regression model that included CSF levels of both *t*-tau and Aβ. Regression investigating the relationship between CSF *p*-tau level and different-cluster judgments was only marginal [*F*_(1, 29)_ = 3.61; *p* = 0.068]. These findings suggest that judgments of narrative organization may be associated with AD-related biomarkers in LBD CSF.

### Imaging analyses

GM atrophy in LBD is illustrated in Figure 2A, and Table 2 lists the anatomic location of peaks within atrophic regions. Table 3 lists the number of atrophic voxels in all regions. Significant atrophy relative to controls (*p* < 0.05, FWE-corrected) was most evident in frontal regions bilaterally, but significant atrophy was also seen in parietal and temporal regions.

**Table 3:** **Anatomic distribution of the numbers of voxels in each MRI region of each MRI analysis in the oasis-30 label set**.

**Anatomical region**	**CSF patients vs. controls**		**Low A**β **voxel overlap**		***t*****-tau voxel overlap**	
	**Left**	**Right**	**Left**	**Right**	**Left**	**Right**
Accumbens area	2	0	6	0	0	0
Amygdala	10	25	3	11	0	0
Brain stem	293		0		122	
Caudate	0	30	17	0	160	97
Hippocampus	92	176	16	85	0	16
Pallidum	6	0	8	0	0	0
Putamen	26	20	76	37	60	108
Thalamus proper	335	248	55	54	37	22
Anterior cingulate gyrus	201	163	6	4	0	0
Anterior insula	217	262	84	50	5	2
Anterior orbital gyrus	6	0	13	17	26	6
Angular gyrus	53	164	137	133	0	0
Calc calcarine cortex	3	141	0	83	0	0
Central operculum	204	131	42	40	0	6
Cuneus	0	66	1	68	0	0
Entorhinal area	27	106	13	62	28	0
Frontal operculum	45	55	4	36	0	6
Frontal pole	11	37	77	201	0	0
Fusiform gyrus	87	87	97	115	17	32
Gyrus rectus	78	21	19	28	15	1
Inferior occipital gyrus	118	92	293	139	0	11
Inferior temporal gyrus	328	241	216	197	17	37
Lingual gyrus	0	58	11	167	3	21
Lateral orbital gyrus	56	17	4	6	0	0
Middle cingulate gyrus	17	28	0	27	0	0
Medial frontal cortex	35	27	8	5	0	0
Middle frontal gyrus	85	95	242	221	19	16
Middle occipital gyrus	186	43	169	97	0	42
Medial orbital gyrus	63	51	20	15	30	11
Postcentral gyrus medial segment	0	0	0	0	0	0
Precentral gyrus medial segment	0	0	0	38	0	0
Superior frontal gyrus medial segment	36	131	5	68	0	0
Middle temporal gyrus	703	691	221	335	0	44
Occipital pole	130	152	123	61	0	0
Occipital fusiform gyrus	5	9	5	30	16	20
Opercular part of the inferior frontal gyrus	28	70	71	32	0	33
Orbital part of the inferior frontal gyrus	61	57	0	1	0	0
Posterior cingulate gyrus	0	34	31	39	14	0
Precuneus	1	248	32	230	0	0
Parahippocampal gyrus	3	9	1	27	0	15
Posterior insula	174	159	50	95	65	49
Parietal operculum	110	68	0	0	17	27
Postcentral gyrus	110	0	20	26	0	0
Posterior orbital gyrus	13	41	15	10	0	0
Planum polare	73	7	2	1	8	8
Precentral gyrus	132	5	122	41	0	5
Planum temporale	115	49	3	15	0	12
Subcallosal area	31	33	20	20	0	0
Superior frontal gyrus	6	64	236	248	24	16
Supplementary motor cortex	1	0	16	44	0	0
Supramarginal gyrus	308	95	286	112	0	22
Superior occipital gyrus	20	56	108	118	0	0
Superior parietal lobule	0	84	132	128	0	0
Superior temporal gyrus	409	314	124	239	0	25
Temporal pole	242	124	67	133	14	0
Triangular part of the inferior frontal gyrus	173	72	104	66	0	0
Transverse temporal gyrus	58	47	2	9	0	7
Parietal operculum	0	0	0	0	0	0

Table 2 summarizes areas where regression indicates a significant relationship between the critical different-cluster judgments and significant atrophy. Implicated regions included left inferior frontal, right inferior frontal, left dorsolateral frontal, and left medial parietal GM. These findings suggest that narrative organization may be related to GM atrophy in LBD.

We also contrasted atrophy in patients with low-Aβ compared to normal-Aβ, illustrated in Figure 2B. Table 3 summarizes the overlap between atrophic voxels and voxels associated with low-Aβ. This analysis identified regions of significant atrophy associated with low-Aβ in dorsolateral and inferior frontal regions bilaterally, as well as temporal, parietal, and striatal regions. Figure 2B and Table 2 show the overlap between regions associated with low-Aβ and ROIs implicated in narrative organization. Regions with significant GM atrophy associated with low Aβ and that are also related to narrative judgments included left dorsolateral frontal, left inferior frontal, and right inferior frontal areas.

As illustrated in Figure 2C and summarized in Table 3, a regression analysis relating GM areas to CSF *t*-tau included left dorsolateral frontal, left inferior frontal, right inferior frontal, right dorsolateral frontal, and striatal regions. Figure 2C and Table 2 show that regions related to CSF *t*-tau that overlap with ROIs implicated in narrative organization included left dorsolateral frontal and left anterolateral temporal areas.

## Discussion

Narrative comprehension is essential to safety and quality of life in LBD (Kempster et al., 2010; Rosenthal et al., 2010). We found that narrative organization is more impaired in mildly demented compared to non-demented patients with LBD, emphasizing their risk for functional impairment. This effect could not be fully explained by overall dementia severity as measured by MMSE. Narrative organization was related to dorsolateral and inferior frontal brain regions as well as anterolateral temporal regions. These regions were also associated with CSF levels of Aβ and *t*-tau, surrogate markers of AD pathology that is often present in LBD. Although it is beyond the scope of this study to validate the anatomic distribution of AD-related pathology in LBD, others have shown Aβ and tau pathology in these frontal and temporal regions (Irwin et al., 2012a; Howlett et al., 2015). These findings suggest that narrative comprehension is impaired in LBD, and we infer from behavioral-MRI-CSF correlations that this deficit may be due in part to the accumulation of pathologic amyloid and tau in an anatomic network centered in the frontal lobe. We discuss these findings below.

Cognitive difficulties are common in LBD (Aarsland et al., 2003; Emre et al., 2007; Postuma et al., 2015; Weintraub et al., 2015). While deficits on standard neuropsychological measures have been demonstrated in these patients (Hessen et al., 2016), here we focused on functional needs often mediated by conversational exchanges that are most effective when organized rather than vague and tangential. Previous work has shown a deficit in LBD patients' organization of narrative expression (Ash et al., 2011), even though these patients are not aphasic. This deficit was related in part to executive difficulty. In the present study of narrative comprehension, all materials were grammatically simple and semantically familiar, suggesting that a linguistic deficit could not fully explain their impairment, and motor difficulty was unlikely to have contributed to this untimed comprehension task. A deficit in ordering events could not easily explain performance since all event pairs—both within-cluster and different-cluster—were consecutively ordered with good accuracy. Nevertheless, we found that mildly demented LBD patients are more impaired judging events in different clusters compared to events in the same cluster, consistent with difficulty appreciating narrative organization.

LBD performance was related to a specific anatomic network of GM atrophy. Converging evidence from fMRI activation studies in healthy adults and neuroanatomic regressions in FTD patients implicated a neural network centered in frontal cortex for processing narrative organization (Farag et al., 2010). We extracted ROIs from this study, and found significant effects relating impaired narrative organization to GM atrophy in this anatomic network in LBD as well. Other work has demonstrated the contribution of dorsolateral prefrontal regions to the organization of complex material (Koechlin and Jubault, 2006) and the role of inferior frontal regions in cognitive control over linguistic materials (Friederici et al., 2005). Left anterolateral temporal GM may play a role in language comprehension (Patterson et al., 2007), and parietal cortex has been implicated in perspective-taking in Theory of Mind assessments (Saxe and Kanwisher, 2003). Interference with the functioning of this anatomic network occurs in other conditions as well such as frontotemporal degeneration, but this anatomic network apparently is not degraded in patients with AD (Farag et al., 2010).

Clinical-pathological studies have related synuclein pathologic burden to cognitive difficulty in LBD (Howlett et al., 2015). Moreover, many LBD patients have Aβ and tau pathology that is seen in AD (Braak and Braak, 1990; Irwin et al., 2012a, 2017). Lower CSF Aβ has been related to impaired cognition in LBD (Montine et al., 2010; Siderowf et al., 2010; Gomperts et al., 2013; Bäckström et al., 2015). Cognitive difficulty in these studies was judged clinically or based on a non-specific survey of dementia, but confounds such as age may cloud the interpretation of the role of CSF analytes in non-specific dementia measures. Thus, it is crucial to relate CSF analytes such as Aβ to more detailed measures of cognition such as narrative organization. Since these cognitive tasks have regional anatomic specificity, the contribution of CSF analytes to cognition can be validated by another measure such as the anatomic distribution of disease using MRI. Here, we provide converging evidence for the role of CSF Aβ in narrative organization that is also associated with MRI atrophy.

We used a validated CSF cutoff of 192 pg/ml (Shaw et al., 2009) to identify LBD patients with pathologic Aβ accumulation, and compared atrophy in these patients with GM in LBD with normal CSF Aβ. Patients with low Aβ had significant narrative organization difficulty. Moreover, areas of GM atrophy associated with reduced CSF Aβ overlapped with dorsolateral and inferior frontal ROIs previously implicated in narrative organization. Aβ may accumulate in LBD when first presenting clinically and this may have clinical consequences early in disease and before dementia (Gomperts et al., 2013; McMillan and Wolk, 2016). Since Aβ accumulation may contribute to cognitive difficulty in all disease stages and not exclusively in dementia, and since some of our non-demented patients had low CSF Aβ, this may explain in part why we failed to find a correlation between MMSE and narrative organization.

The relationship between CSF tau and cognition in LBD is less clear (Hall et al., 2016; Kang et al., 2016; Terrelonge et al., 2016). We found that *t*-tau is related to narrative organization difficulty. Regression showed that GM atrophy related to CSF *t*-tau overlapped with frontal and anterolateral temporal ROIs implicated in narrative organization. Previous inconsistencies relating tau to cognition may be due in part to a mismatch between the task assessing cognition and the anatomic focality of tau accumulation. Another possible confound is the high correlation between CSF tau and α-syn in LBD (Hall et al., 2016). This may be due in part to the release of both *t*-tau and α-syn in neuronal disease from adjacent axonal and synaptic loci, respectively, and additional work is needed to determine whether tau has an independent contribution to cognitive disorders of LBD. Previous work related *p*-tau to executive deficits (Hall et al., 2016), although the present study found only a borderline effect for *p*-tau. Additional work is needed to establish the role of various forms of tau in cognitive difficulty in LBD.

Several shortcomings should be kept in mind when considering our findings. While we studied a deeply endophenotyped subset of cases, our cohort was small and additional work is needed to confirm our findings in a larger group. We related CSF Aβ and tau levels to anatomic areas of GM atrophy statistically, and additional work with Aβ and tau PET radioligands can assess the role of these and other anatomic regions associated with pathology more directly. With these caveats in mind, our findings are consistent with the hypothesis that LBD patients have difficulty appreciating narrative organization, and this may be due in part to the accumulation of AD-related Aβ and tau pathology in frontal brain regions that support narrative organization. Our findings suggest that narrative comprehension performance may serve as an inexpensive screen for the presence of Aβ and tau pathology in LBD, and that disease-modifying treatments for these pathologies may stabilize or improve performance on important functional tasks such as narrative comprehension.

## Ethics statement

This study was carried out in accordance with the recommendations of the University of Pennsylvania Institutional Review Board. All subjects participated in a written informed consent procedure in accordance with the Declaration of Helsinki. This protocol was approved by the University of Pennsylvania Institutional Review Board.

## Author contributions

MG: Study development and design, data collection, data analysis, manuscript preparation, obtaining funding. DI: Data collection, data analysis, manuscript revision. CJ: Data analysis. AH: Data collection. SA: Data analysis. KR: Data collection. DW: Manuscript revision. CM: Data analysis, manuscript revision.

## Funding

This work was supported in part by NS053488, AG038490, AG017586, NS043503, NS088341 from the National Institutes of Health.

### Conflict of interest statement

The authors declare that the research was conducted in the absence of any commercial or financial relationships that could be construed as a potential conflict of interest.
